# Histone deacetylase inhibitors blocked activation and caused senescence of corneal stromal cells

**Published:** 2008-12-30

**Authors:** Qingjun Zhou, Yao Wang, Lingling Yang, Ye Wang, Peng Chen, Yiqiang Wang, Xiaoguang Dong, Lixin Xie

**Affiliations:** Shandong Provincial Key Lab of Ophthalmology, Shandong Eye Institute, Qingdao, China

## Abstract

**Purpose:**

Corneal myofibroblasts differentiated from activated corneal stromal cells are the major cellular sources of extracellular matrix synthesis for the repair of corneal injury. In this study, the effects of histone deacetylase (HDAC) inhibitors on the activation, proliferation, migration and senescence of corneal stromal cells were evaluated.

**Methods:**

Primary human and mouse corneal stromal cells were harvested by sequential digestion with dispase and collagenase, and cultured in DMEM/F-12 media under serum-free (keratocytes), serum- (corneal fibroblasts) and TGFβ1-supplemented (corneal myofibroblasts) conditions. The responses of corneal stromal cells to HDAC inhibitors were characterized by cDNA microarray, real time PCR, immunocytochemistry and western blot analysis. The effects of HDAC inhibitors on corneal fibroblast proliferation, cell cycle distribution, migration and senescence were also assessed in vitro.

**Results:**

Fetal bovine serum and TGFβ1 activated the transdifferentiation of corneal stromal cells into fibroblasts and myofibroblasts, indicated by cell spreading, renewed assembly of actin filaments and enhanced expression of extracellular matrix components, all of which were suppressed by the addition of HDAC inhibitors. HDAC inhibitors inhibited the proliferation of corneal fibroblasts by decreasing the proportion in the S-phase and increasing the proportion in the G0/G1 and G2/M cell cycle checkpoints. HDAC inhibitors showed a dose-dependent inhibitory effects on the migration of corneal fibroblasts. In addition, HDAC inhibitors induced the senescence of corneal myofibroblasts as shown by enhanced staining of β-galactosidase and upregulated expression of *p16^ink4a^*.

**Conclusions:**

HDAC inhibitors may affect corneal stromal cells by inhibiting myofibroblastic differentiation, cell proliferation, migration and by inducing cell senescence. Thus, this has implications for future studies in the development of promising drugs in the prevention or treatment of corneal haze and scar formation.

## Introduction

Corneal keratocytes are the major cellular components in the corneal stroma. Normally, the cells remain quiescent and synthesize key extracellular matrix (ECM) components, maintaining their turnover in corneal stroma. During corneal wound healing, except for the anterior keratocytes undergo apoptosis, the corneal stromal cells in the posterior stroma are activated and undergo the transformation into corneal fibroblasts and myofibroblasts after migration to the wound site, which were characterized by the expression of α-smooth muscle actin (α-SMA), the synthesis of large amounts of ECM components, and the increased proliferation and acquisition of contractile properties [1]. Recent studies have shown that TGFβ1 increased corneal stromal cell proliferation and was the only isoform of TGFβs to promote the transdifferentiation. From a therapeutic point of view, molecules that can block the fibrogenic effects of TGFβ1, collagen synthesis, fibroblast proliferation are of great clinical interests [2]. In mammalian cells, the state of acetylation or deacetylation of core nucleosomal histones is regulated by the interactions between histone acetyltransferases (HATs) and histone deacetylases (HDACs), which play important roles in the regulation of gene expression by affecting chromatin structure [3]. Currently, known inhibitors of histone deacetylases, such as sodium butyrate (NaBu), trichostatin A (TSA) and valproic acid (VPA), can induce the hyperacetylation of histones and nonhistone protein substrates, such as NF-kappaB, HIF-1α, etc [3,4]. As reported in recent studies, HDAC inhibitors exert antifibrotic effects by suppressing the main features of TGFβ-induced myofibroblastic differentiation of cultured hepatic stellate cells [5], pancreatic stellate cells [6,7] and skin fibroblasts [8,9]. In vivo studies revealed that TSA prevented the dermal accumulation of extracellular matrix in bleomycin-induced fibrosis [10]. Corneal stromal cells shared similar roles and characteristics with hepatic stellate cells and skin fibroblasts [1,11-14], so we hypothesized that epigenetic regulations may be involved in the differentiation of corneal keratocytes into fibroblasts and myofibroblasts. Recently, Horswill reported that DNA methylation was involved in the regulation of maspin expression in the conversion of keratocytes to fibroblasts [15]. However, the effects of HDAC inhibitors on the activation and differentiation of corneal stromal cells remain elusive.In the present study, we found that the inhibitors of histone deacetylases inhibited the activation and transdifferentiation of corneal stromal cells, suppressed the proliferation of corneal fibroblasts by decreasing the proportion of cells in S-phase, and inhibited the migration of corneal fibroblasts in a dose-dependent manner. In addition, HDAC inhibitors induced the senescence of corneal fibroblasts as shown by the enhanced staining of β-galactosidase and the upregulated expression of *P16^INK4A^*. The above results demonstrated that epigenetic modifications may play important roles in the different phases of corneal wound healing. Further investigations into the molecular mechanisms may reveal new therapeutic strategies for the prevention or treatment of corneal haze and scar formation.

## Methods

### Isolation of human and murine corneal stromal cells

For the isolation of human corneal stromal cells, human corneal tissues were obtained from the Eye Bank at Shandong Eye Institute after the central corneal buttons were removed for clinical application. Corneal epithelium and endothelium were removed by digestion with 50 mg/ml dispase II (Roche) overnight at 4 °C. The corneal stroma was cut into pieces and incubated 6–8 h at 37 °C in DMEM/F-12 medium containing 1.25 mg/ml collagenase (Invitrogen), until the tissue smeared onto the dish bottom [16,17]. For the isolation of mouse corneal stromal cells, mice (C57BL/6) eyes were enucleated with forceps after euthanasia, and corneal stromal cells were isolated as described above. The acquired human and mouse keratocytes were suspended in DMEM/F-12 medium supplemented with 20 mM HEPES, ITS (5 μg/ml insulin, 5 μg/ml transferrin, and 5 ng/ml sodium selenite), 100 U/ml penicillin, and 100 mg/ml streptomycin to maintain their quiescent status [18,19].

### Cell culture and treatment

Freshly isolated corneal stromal cells were divided into 5 groups as followed, The 1st group cells were plated as primary quiescent keratocytes on cell culture dishes in DMEM/F-12 medium containing HEPES, ITS and antibiotics as described above. The 2nd group cells were cultured in the DMEM/F-12 medium containing 10% fetal bovine serum (FBS, Gibco) for the activation of corneal fibroblasts. The 3rd group cells were treated with 1ng/ml TGFβ1 (R&D) for 3 to 5 days in the presence of 10% FBS [11,17] for the myofibroblastic transdifferentiation. To examine the effects of HDAC inhibitors (NaBu and TSA) on the activation and transdifferentiation of corneal stromal cells, the 4th and 5th group cells were treated with FBS or TGFβ1 in the presence of HDAC inhibitors. The responses of corneal stromal cells to HDAC inhibitors were characterized by cDNA microarrays, real time PCR, immunocytochemistry and western blot analysis. Cell proliferation, cell cycle distribution and migration were determined by CFSE or PI staining and FACS analysis and wound healing assay as described below.

### cDNA microarray

The 3rd passage corneal fibroblasts were divided into three groups as follows: untreated corneal fibroblasts were considered as the control group, corneal fibroblasts treated with TGFβ1 for four days in the presence of 10% FBS were considered as the myofibroblastic differentiation group, and corneal fibroblasts treated with TGFβ1 and 400 nM TSA for four days were considered as the myofibroblastic differentiation inhibition group. All three populations of corneal fibroblasts were used for cDNA microarrays as described previously [20]. In brief, total RNAs were extracted using Trizol (Invitrogen, Gaithersburg, MD), precipitated with isopropanol, and purified using the NucleoSpin RNA clean-up Kit (MACHEREY-NAGEL, Germany). The quality and quantity of total RNA samples were determined by formaldehyde-denatured agarose gel electrophoresis and spectrophotometry, respectively.

For the cDNA microarray, RNA was reverse-transcribed to cDNA and, during this process, a T7 sequence was introduced into the cDNA. The T7 RNA polymerase-driven RNA synthesis was used for the preparation and labeling of cRNA with Cy3-dCTP and Cy5-dCTP. The labeled products were purified with the PCR NucleoSpin Extract II Kit. An equal amount of Cy3 and Cy5 labeled probes were mixed and used for hybridization to one 35k human genome array (CapitalBio Corp., Beijing, China), following the protocol provided by the manufacturer. The hybridization signals were acquired by using the LuxScan 10KA microarray laser scanner (CapitalBio) and analyzed using the LuxScan™ 3.0 Software. Specifically, the Linear & Lowess method and the rank consistency filter were used for normalization of the features. The rank consistency filter selects features that fall within the central tendency of the data by observing consistent trends between the red and green channels. To be counted as a valid feature, the feature has to pass four criteria: 1) they are positive and significantly different compared with just the background, 2) the signals are uniform in the spot, 3) the signals are not saturated, and 4) they are not population outliers in either channel. If the features failed in any one of the four criteria, they were flagged and excluded from further analysis.

### Real time polymerase chain reaction

Total RNAs were extracted from corneal fibroblasts, TGFβ1-treated corneal fibroblasts and TGFβ1 and TSA co-treated corneal fibroblasts using NucleospinRNA Kits (BD Biosciences, Palo Alto, CA). cDNAs were synthesized from total RNA using an AMV first-strand cDNA synthesis kit (BBI, Toronto, Canada), according to the manufacturer’s instructions. Quantitative real time PCR was performed using Taqman reagents and the Applied Biosystems 7500 Real Time PCR System (Applied Biosystems, Foster City, CA) according to the instructions of the manufacturer. The specific primers and probes used in this assay are listed in Table 1. Cycling conditions were 10 min at 95 °C followed by 40 two-step cycles (15 s at 95 °C and 1 min at 60 °C). The quantification data were analyzed with the SDS System Software (Applied Biosystems) using GAPDH as an internal control.

**Table 1:** Primers used for real time PCR.

**Gene**	**Forward primer**	**Reverse primer**	**Probe**	**Product (bp)**
α-SMA	GGTGACGAAGCACAGAGCAA	CAGTTGGTGATGATGCCATGTT	AGAGGAATCCTGACCCTGAAGTACCCGA	74
Collagen I	TTGTGCGATGACGTGATCTGT	TTGGTCGGTGGGTGACTCTG	CGAGGGCGAGTGCTGTCCCGT	111
Collagen IV	GCAAACGCTTACAGCTTTTGG	GGACGGCGTAGGCTTCTTG	CGCCACCATAGAGAGGAGCGAGATG	69
p21 ^waf1/cip1^	CAGACCAGCATGACAGATTTC	TTAGGGCTTCCTCTTGGAGA	ACCACTCCAAACGCCGGCTG	66
p27 ^kip1^	CCTCCTCCAAGACAAACAGC	CATTCAGAGCGGGATTATCTTT	TCGAGTTCCTGACAAGCCACGC	89
p16 ^ink4a^	AAGGTCCCTCAGACATCCC	TGTAGGACCTTCGGTGACTG	TCCGGAGGTTTCTCAGAGCCTCTC	90
GAPDH	ATGCTGGCGCTGAGTACGT	AGCCCCAGCCTTCTCCAT	TGGAGTCCACTGGCGTCTTCA	65

### Immunocytochemistry and western blot analysis

For immunocytochemistry, cells were fixed with 4% paraformaldehyde (PFA) followed by ice-cooled methanol. After blocking with 5% normal goat serum, the samples were incubated with FITC-conjugated phalloidin (Alexis), or with primary rabbit anti-α-SMA and anti-collagen I, overnight at 4 °C. Following three washes with PBS, the cells were incubated with FITC or rhodamine-conjugated goat anti-rabbit secondary antibodies and counterstained with DAPI. Cell staining was examined under a Nikon confocal laser-scanning microscope. Antigen-unrelated primary antibody was used for negative control staining.

For western blot analysis, total proteins were extracted using Active Motif nuclear and cytoplasm extract kit, according to manufacturer’s instructions. Briefly, the collected cells were lysed in the hypotonic buffer containing detergent and centrifuged for 30 s at 14,000x g. The supernatants were collected and stored at −80 °C, until ready for use. Samples were run on 10% SDS–PAGE gels for 1 h at 160 V and then transferred to nitrocellulose. The blots were blocked in 5% non-fat dry milk dissolved in TBST for at least 1 h, then incubated with rabbit anti α-SMA and anti collagen I in TBST for 1 h at room temperature. The blots were washed three times, each time with 10 ml of TBST, and then incubated for 1 h at room temperature with a horseradish peroxidase-conjugated secondary antibody (Amersham Biosciences). Finally, the blots were washed three times, each time with 10 ml of TBST, and were visualized via enzyme-linked chemiluminescence using the ECL kit (Chemicon).

### Cell proliferation analysis

CFSE staining was performed as previously described, to evaluate the proliferation of corneal fibroblasts after the treatment with NaBu or TSA [21]. Briefly, corneal fibroblasts were washed with PBS for 3 times and resuspended in pre-warmed PBS containing 2 µM CFSE dye (CFDA SEM Cell Tracer kit; Molecular Probes) for 15 min at 37 °C. Cells were then washed and incubated with fresh PBS for an additional 30 min to allow complete modification of the probe before adoptive cell transfer. As described above, the labeled cells were cultured in the presence or absence of HDAC inhibitors for 3 days and analyzed by flow cytometry.

### Cell cycle analysis

Flow cytometry was performed to measure the cell cycle distribution of HDAC inhibitors treated corneal fibroblasts [22]. For the staining of cellular DNA, the cells were fixed in ice-cold 70% ethanol and incubated in PBS containing 30 μg/ml propidium iodide (PI) and 0.3 mg/ml RNase A. The measurements were made with a Becton Dickinson FACS Calibur machine, adapted for excitation with a 488 nm argon laser, and 582/42 nm band-pass filter for detecting PI emission. A total of 20,000 cells were collected by FACS and analyzed using the CellQuest 3.1 software (Becton Dickinson). On each occasion, at least three samples of each treatment were analyzed.

### Cell migration analysis

Corneal fibroblast migration was evaluated by wound healing assay according to previous descriptions [23]. Briefly, corneal fibroblasts were seeded on 24 well tissue culture plates. When the cells reached 90% confluence, they were wounded with a micropipette tip and marked as the injury lines. The cultures were rinced with culture medium to remove detached cells, and incubated with growth medium containing different concentrations of TSA or solvent control for 24 h. Digital images of wound closure were obtained and used for quantitative assessment of migration by counting the number of cells that migrated beyond the injury lines in five views. Each assay was conducted at least triplicate.

### Senescence-associated β-galactosidase staining assay

Cell senescence was examined with the senescence-associated β-galactosidase (SA-β-gal) staining Kit (Beyotime, Haimen, China), according to the manufacturer’s instructions. Briefly, the cells were washed twice with PBS and fixed for 15 min in room temperature, then washed again to remove the fixing solution, incubated in the SA-β-gal staining solution for overnight at 37 °C and viewed under the Nikon microscope. The different staining densities were separately calculated by computerized image analysis, using imagepro plus software (Media Cybernetics, Silver Spring, MD). The cells of positive control group were incubated daily in the presence of 100µM H_2_O_2_ for 1 h for 3 days. The cells were washed with PBS and incubated in the normal culture medium for 24 h. After the last treatment, the cells were allowed to recover for 3 days before the SA-β-gal staining.

### Statistical analysis

The data are presented as means±SD. The differences between control and experimental conditions were tested with Student’s *t*-tests. A p value of less than 0.05 was considered to be statistically significant.

## Results

### HDAC inhibitors suppressed TGFβ1 mediated myofibroblastic differentiation

Human and mouse corneal stromal cells were exposed to TGFβ1 and/or HDAC inhibitors for 3–5 days to examine the potential effects of HDAC inhibitors on the differentiation of TGFβ1-induced corneal keratocytes into myofibroblasts. Morphological changes were assessed and the relative amounts of α-SMA and collagens transcript and protein levels were evaluated by real time PCR, western blot and immunocytochemistry, respectively.

### Morphological analysis

**Figure 1:**
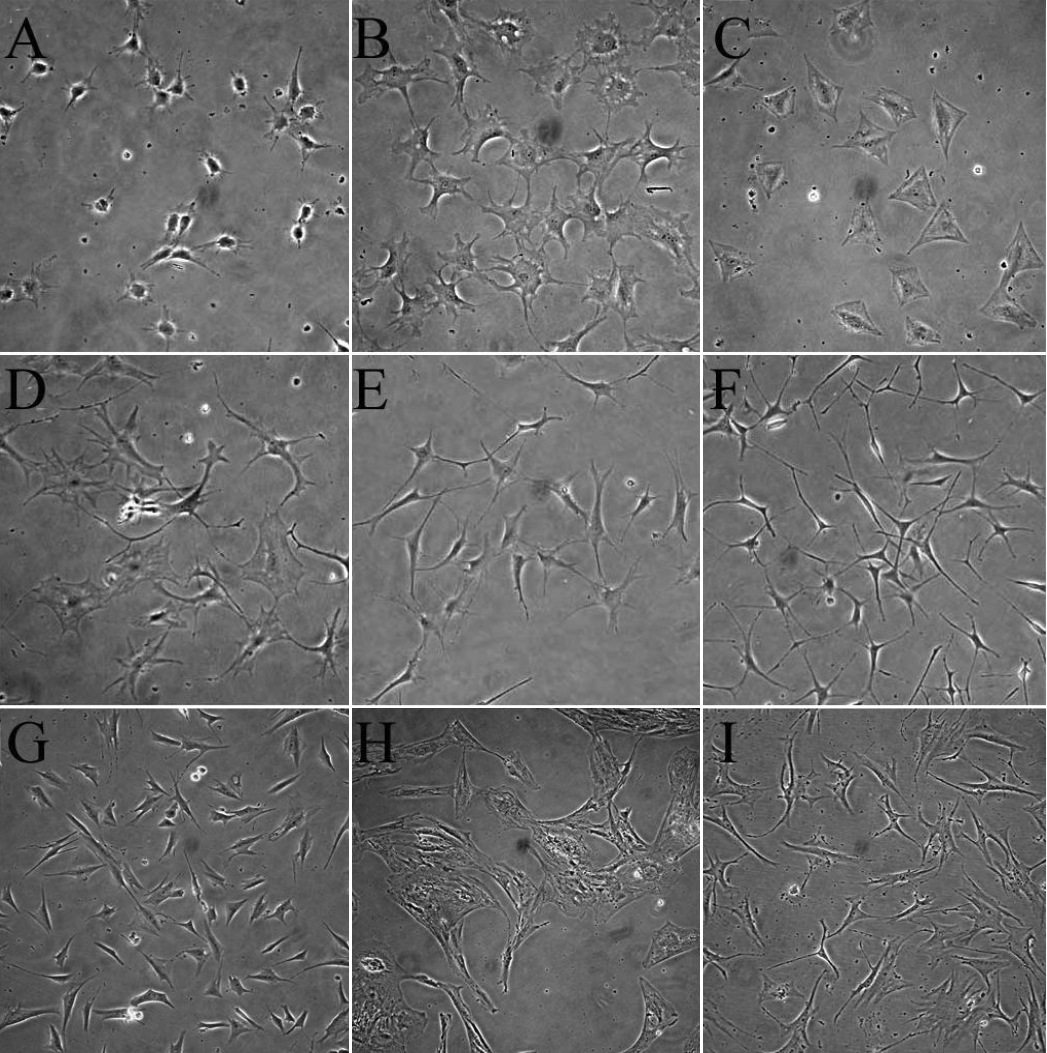
Trichostatin A prevented morphological changes of TGFβ-mediated myofibroblastic differentiation of corneal fibroblasts. Freshly isolated mouse corneal keratocytes exhibited a dendritic-like morphology when cultured in the serum-free medium (**A**). Serum (10% FBS) and TGFβ (1 ng/ml) induced rapid changes of cellular morphologies from dendritic to spreading and caused cells to finally assume myofibroblastic morphology (**B**, **C**). The addition of TSA suppressed the differentiation mediated by fetal bovine serum (**D**) and TGFβ (**E**, **F**), and the inhibition was dose-dependent as shown in (**E**; 100 nM TSA) and (**F**; 400 nM). Human quiescent corneal keratocytes (**G**), TGFβ induced the differentiated myofibroblasts (**H**), and 400 nM TSA treated corneal fibroblasts (**I**) exhibited similar morphological changes. (200X)

In normal corneal stroma, the keratocytes showed stellate morphology with numerous cytoplasmic extensions. Similar to their assumed in vivo morphology, freshly isolated mouse corneal keratocytes exhibited dendritic morphology when cultured in the serum-free medium (Figure 1A). Serum and TGFβ induced rapid changes of cellular morphologies from dendritic to spreading, and finally to myofibroblastic morphology (Figure 1B,C). However, both the FBS- and TGFβ-induced morphological changes of mouse corneal keratocytes were suppressed by TSA (Figure 1D-F), which preserved the dendritic shapes and long cytoplasmic extensions. The inhibitions of TSA on the morphologic changes occurred in a dose-dependent manner, as shown in Figure 1E (100 nM TSA) and Figure 1F (400 nM). For the human keratocytes, 400 nM TSA also inhibited the myofibroblastic transdifferentiation of corneal stromal cells induced by TGFβ in the presence of 10% FBS (Figure 1G-I).

### Immunocytochemistry analysis

**Figure 2:**
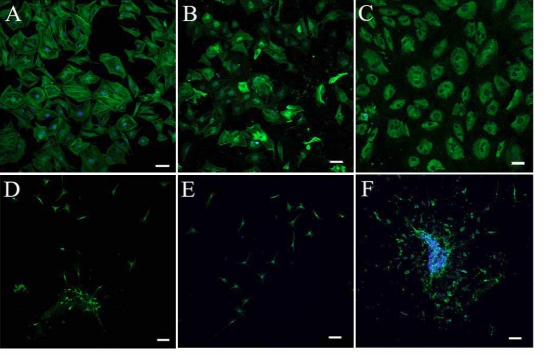
Trichostatin A suppressed the myofibroblastic differentiation of mouse corneal stromal cells induced by TGFβ1. Corneal fibroblasts treated with 1 ng/ml TGFβ1 for 4–5 days exhibited enhanced staining with FITC-conjugated phalloidin (**A**), α-SMA (**B**), and collagen I (**C**). However, the keratocytes treated with TGFβ1 in the presence of 400 nM TSA remained small with dendritic-like morphology and weak or only minimal staining of phalloidin (**D**), α-SMA (**E**), and collagen I (**F**). Bar, 50 µm.

Immunocytochemistry analysis was used to confirm the myofibroblastic differentiation of mouse corneal stromal cells induced by TGFβ1. As shown in Figure 2A, TGFβ1-treated cells exhibited spreading morphology and enhanced staining with FITC-conjugated phalloidin, which showed the organization of F-actin into prominent intracellular bundles that was consistent with the formation of stress fibers. Staining with antibodies to α-SMA and collagen I also revealed an enhanced expression of corneal fibroblasts induced by 1ng/ml TGFβ1 for four days. However, when the freshly isolated mouse keratocytes were treated with TGFβ1 in the presence of 400 nM TSA, the cells retained their small dendritic-like morphology and weak staining of phalloidin (Figure 2D). Although the staining of collagen I antibody was weakly positive for the corneal stromal cells treated with TGFβ1 and TSA, the cells showed no staining or only minimal staining with antibodies for α-SMA that is specific to differentiated myofibroblasts. In addition, 5 mM NaBu exhibited similar inhibitory effects as 400 nM TSA (data not shown). Therefore, HDAC inhibitors were able to prevent TGFβ1-mediated myofibroblastic differentiation of corneal stromal cells in vitro as assessed by morphological and immunocytochemistry analyses.

### Gene expression analysis

**Figure 3:**
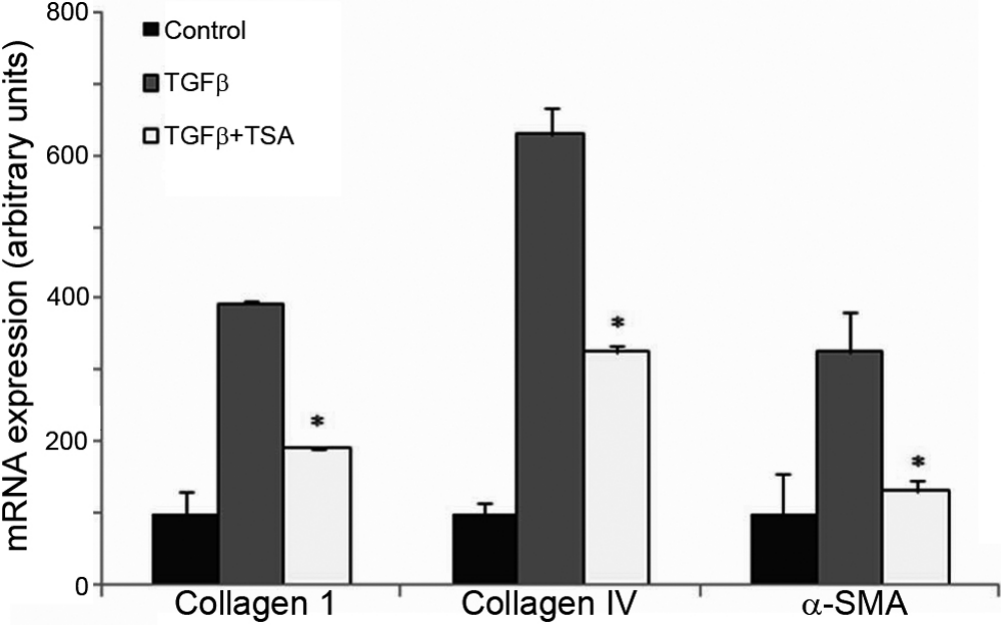
Real time PCR confirmation of the microarray results. Compared with control conditions, stimulation of human corneal fibroblasts with TGFβ1 induced 3.92, 6.34, and 3.35 fold increases in the abundance of collagen I, collagen IV and α-SMA, respectively. Compared with TGFβ treated cells, TSA and TGFβ treatment down-regulated the expressions of collagen I (0.48 fold), collagen IV (0.52 fold) and α-SMA (0.41 fold).

The results of microarrays revealed that the expressions of genes associated with myofibroblastic differentiation of corneal fibroblasts were upregulated markedly after the induction with TGFβ1 for 4 days, mainly for the collagen family, such as collagen I, IV, VII, X, XI, and several members of the actin family, such as α-SMA, γ-enteric smooth muscle actin, α-cardiac actin and α-actinin 1 (Table 2). The addition of 400 nM TSA significantly suppressed the upregulation of the expressions of the genes induced by TGFβ (Table 2). The partial results of the microarrays, including collagen I and IV, α-SMA were further confirmed by real time PCR analysis. Compared with control conditions, the stimulation of human corneal fibroblasts with TGFβ1 induced 3.92, 6.34 and 3.35 fold increases in the abundance of collagen I, collagen IV and α-SMA, respectively. TSA at 400 nM concentration down-regulated the expressions of collagen I (0.48-fold), collagen IV (0.52-fold) and α-SMA (0.41-fold) compared with TGFβ treated cells (Figure 3), which were consistent with the results of the microarray analysis.

**Table 2:** TSA inhibited the normally upregulated expression of fibrotic-related genes induced by TGFβ.

**TGFβ/ control**	**TGFβ+ TSA/TGFβ**	**Description**	**RefSeq**
3.8358	0.3852	Collagen alpha 1(I) chain precursor	NM_152624
8.8109	0.4127	Collagen alpha 1(IV) chain precursor	NM_032812
3.8324	0.6006	Collagen alpha 1(VII) chain precursor	NM_013390
7.1472	0.0319	Collagen alpha 1(X) chain precursor	NM_003725
6.3617	0.7244	Collagen alpha 1(XI) chain precursor	NM_001718
5.8157	0.1892	Actin, aortic smooth muscle	NM_014840
3.4333	0.3078	Actin, gamma-enteric smooth muscle	NM_138431
3.1596	0.5266	Actin, alpha cardiac	NM_001937
2.6436	0.4768	Alpha-actinin 1	NM_000219

### Western blotting analysis

**Figure 4:**
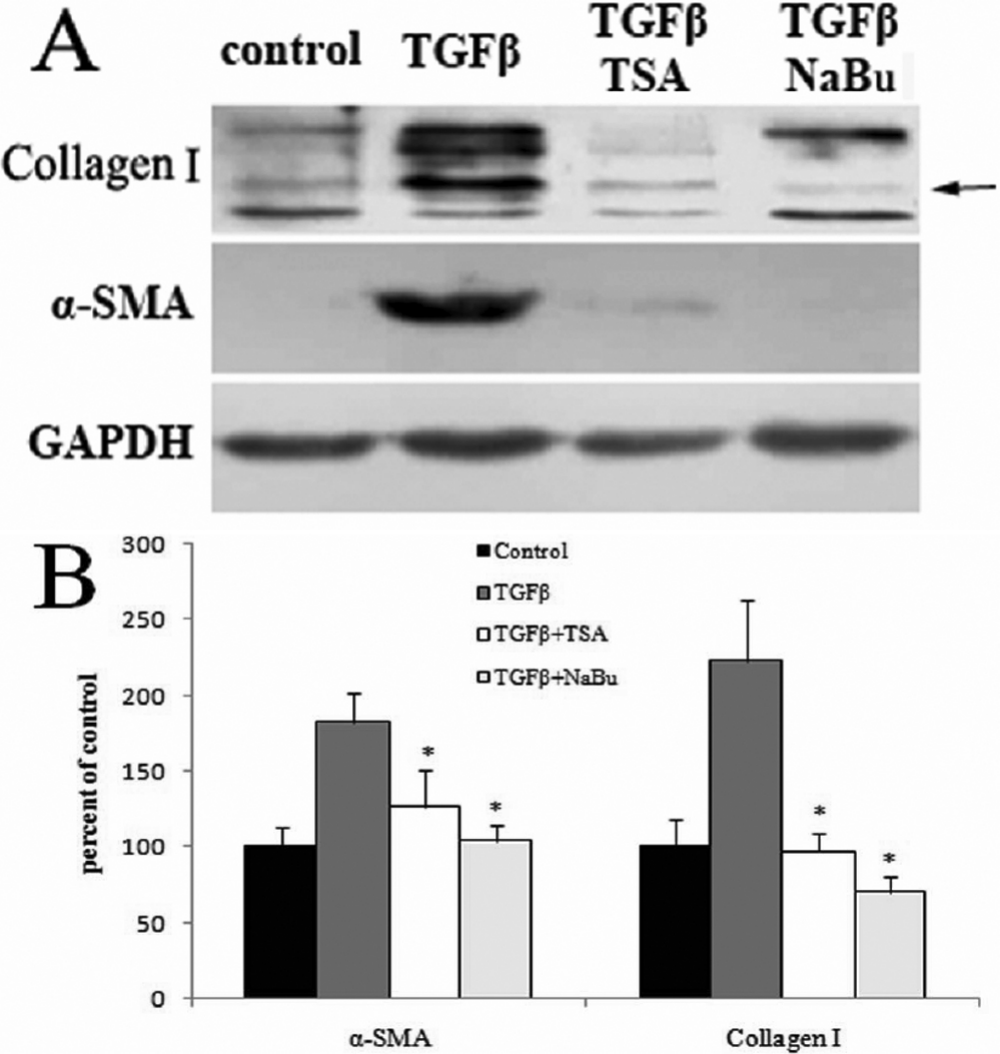
TSA and NaBu suppressed the TGFβ-mediated upregulation in the expression of collagen I and α-SMA at the protein levels. As shown by the analysis (**B**) of western blotting (**A**), TGFβ1 significantly upregulated the protein levels of α-SMA and collagen I by 1.8 and 2.2 fold, respectively, while 400 nM TSA strongly inhibited the synthesis of α-SMA and collagen I by 32% and 57%, respectively, and 5 mM NaBu suppressed the synthesis of α-SMA and collagen I by 43% and 69%, respectively.

For the quantification of protein levels, the effects of TGFβ1 and HDAC inhibitors on the expression of α-SMA and collagen I protein were evaluated by western blotting. The results are shown in Figure 4. TGFβ1 significantly upregulated the protein levels of α-SMA and collagen I by 1.8 and 2.2 fold, respectively, while 400 nM TSA strongly inhibited the synthesis of α-SMA and collagen I by 32% and 57%, respectively, and 5 mM NaBu also suppressed the synthesis of α-SMA and collagen I by 43% and 69%, respectively. The cells treated with HDAC inhibitors alone showed similar expression patterns to the control group (data not shown). In conclusion, TGFβ1 upregulated the expressions of fibrosis-associated factors, and thus promoted the transdifferentiation of corneal fibroblasts into myofibroblasts. TSA and NaBu almost completely inhibited the expressions of fibrosis-related genes, both at the mRNA and protein levels.

### HDAC inhibitors inhibited the proliferation of corneal fibroblasts

**Figure 5:**
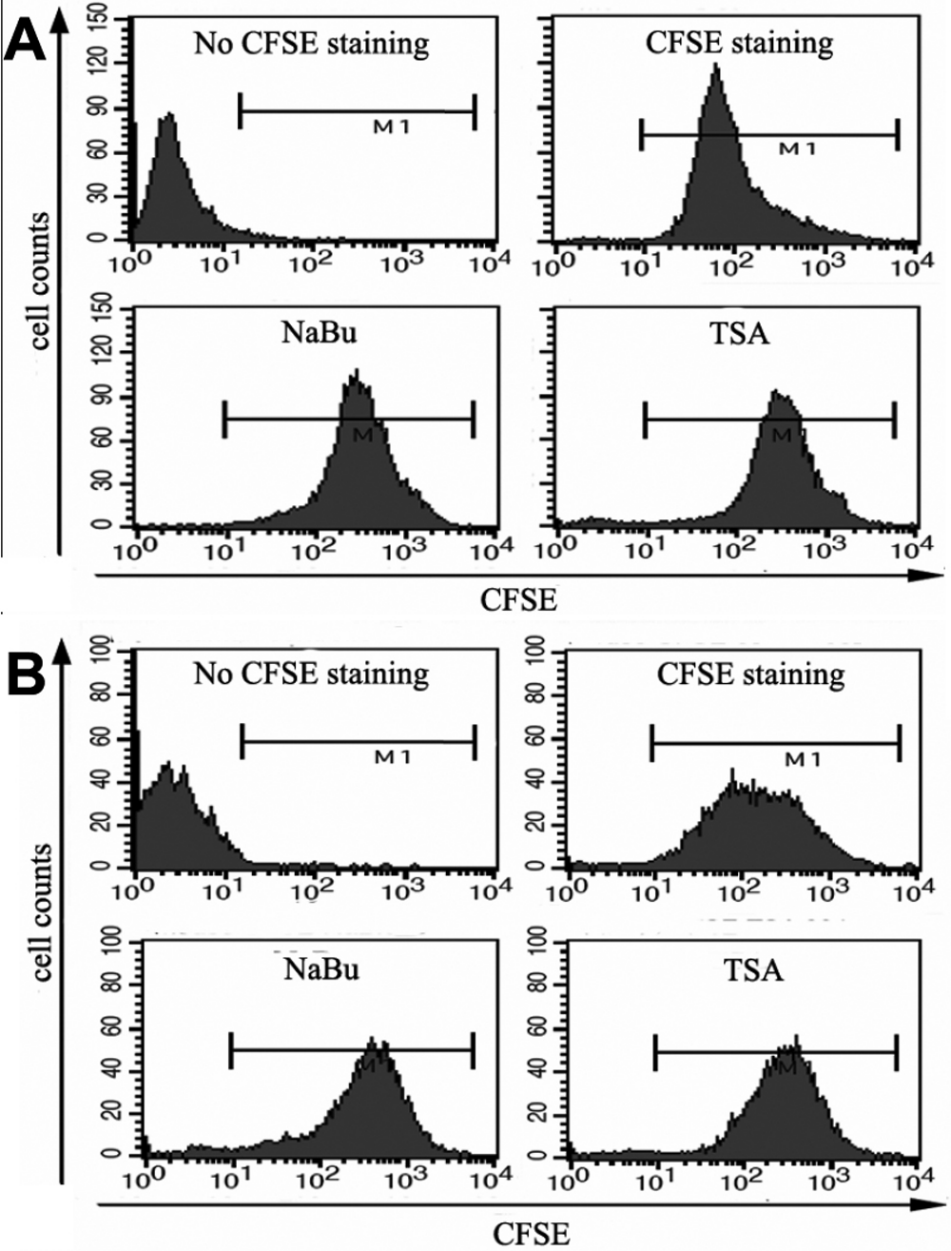
TSA and NaBu inhibited the proliferation of corneal fibroblasts. Human (**A**) or mouse (**B**) corneal fibroblasts were treated with 400 nM TSA or 5 mM NaBu for 3 days. In vitro proliferation assays were performed with the fluorescent dye CFSE and analyzed by FACS. Loss of CFSE fluorescence reflects cellular division.

Carboxy-fluorescein diacetate, succinimidyl ester (CFSE) is a membrane permeable dye used to examine the proliferation of specific lymphocyte subsets [21,22]. During each round of cell division, relative fluorescence intensity of the dye is decreased by half. Here, we used it for the examination of the inhibitory effects of HDAC inhibitors on the proliferation of corneal fibroblasts. Mouse and human corneal fibroblasts were first incubated with CFSE, which passively diffused into cells. Excess dye was washed away and the cells are treated with TSA or NaBu for three days. Corneal fibroblasts showed heterogeneous intensity in the fluorescence, which showed different successive cell generations (data not shown). The results of FACS analysis showed that both of the two HDAC inhibitors inhibited the proliferation of human (Figure 5A) and mouse (Figure 5B) corneal fibroblasts.

### HDAC inhibitors caused cell cycle arrested of corneal fibroblasts

**Figure 6:**
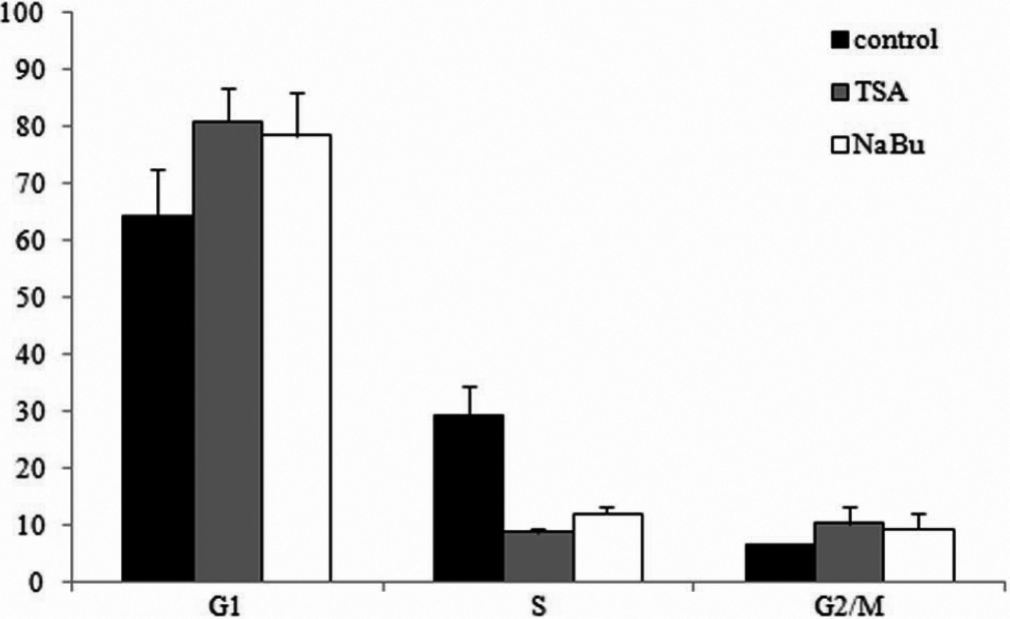
Cell cycle distribution of corneal fibroblasts treated with TSA or NaBu. In normal human corneal fibroblasts, about 64.4%, 6.5% and 29.1% were in the G_0_/G_1_, G_2_/M, and S phase, respectively. After treatment with 400 nM TSA or 5 mM NaBu for 3 days, the proportion of cells in the G_0_/G_1_ phase increased to 80.9% with TSA and to 78.5% with NaBu, the proportion of cells in the G_2_/M phase increased to 10.4% with TSA and 9.4% with NaBu, and the proportion of cells in the S phase reduced to 8.7% with TSA and 12.1% with NaBu.

The results of CFSE staining and FACS analysis revealed that HDAC inhibitors inhibited the proliferation of corneal fibroblasts. As described previously, inhibitory effects of HDAC inhibitors on the proliferation are often caused by cell cycle arrested [3]. Here, we evaluated the effect of TSA and NaBu on the cell cycle profiles of human corneal fibroblasts. As shown in Figure 6, about 64.4% and 6.5% of normal human corneal fibroblasts were in the G_0_/G_1_ and G_2_/M phases, respectively, and 29.1% of the cells were in the S phase. In contrast, after the treatment with 400 nM TSA or 5 mM NaBu for three days, the proportion of cells in the G_0_/G_1_ phase were 80.9% (TSA) and 78.5% (NaBu), the proportion of cells in the G_2_/M phase were 10.4% (TSA) and 9.4% (NaBu), and the proportion of cells in the S phase was reduced to 8.7% (TSA) and 12.1% (NaBu). Taken together, these data suggested that TSA and NaBu reduced the proliferation of corneal fibroblasts, and this was caused by decreasing the proportion of cells in S phase and increasing the proportion of cells in the G_0_/G_1_ and G_2_/M cell cycle checkpoints (Figure 6).

### HDAC inhibitors repressed the migration of corneal fibroblasts

**Figure 7:**
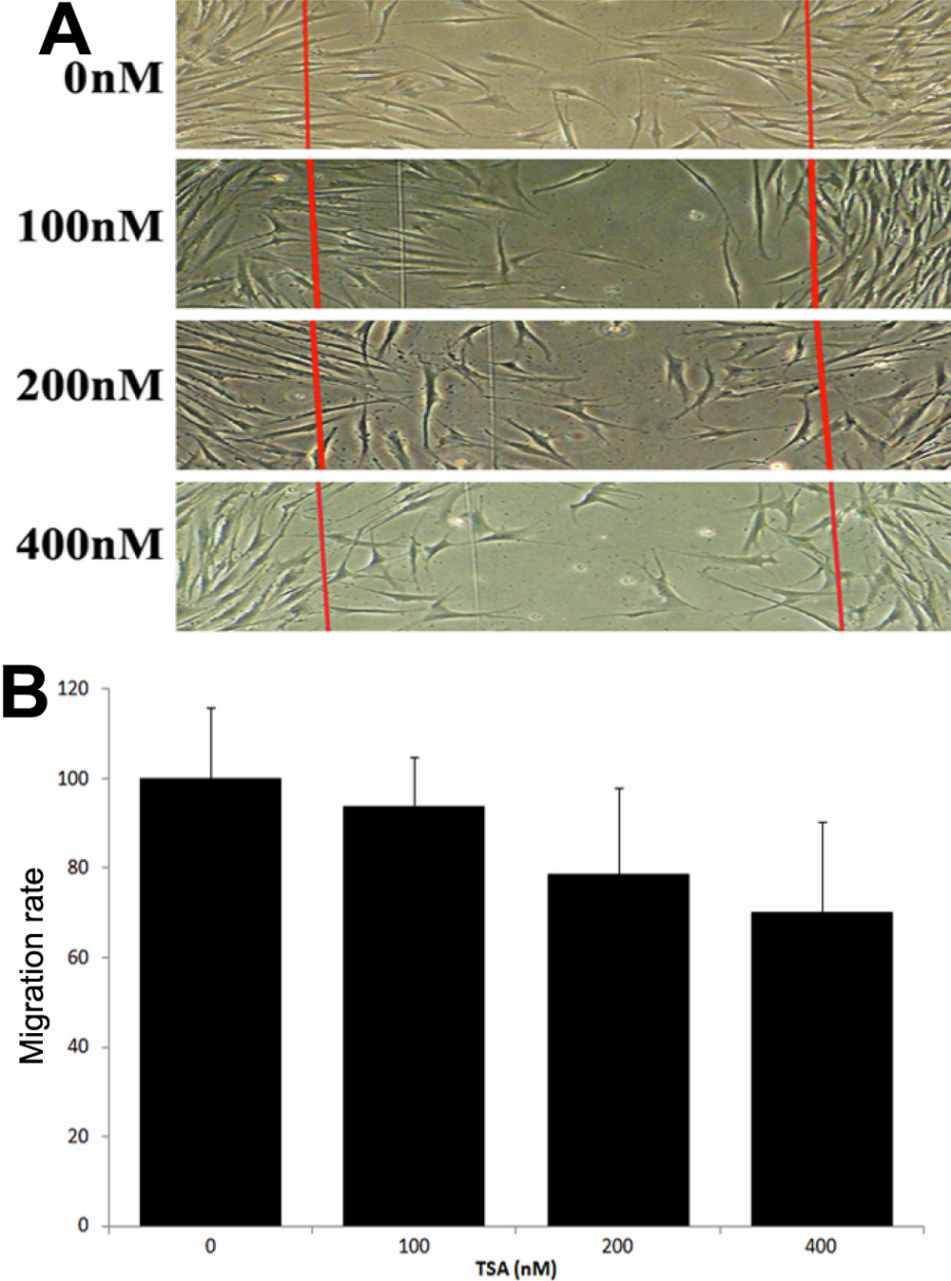
Effects of TSA on wound healing migration of corneal fibroblasts. The wounds were introduced by scraping the cell layers with a pipette tip. **A**: Representative photographs of migrated cells that received either control treatment or different concentrations of TSA. **B**: The number of cells migrated between the injury lines were counted and compared. Migration rate was determined as follows: migration rate=(cell number of TSA group between the injury lines/cell number of control group between the injury lines) x100%.

The migration of corneal fibroblasts plays an important role in the scar formation [1]. Here we evaluated the inhibitory effects of TSA on the migration of corneal fibroblasts using the wound healing assay. The number of cells migrated into the two injury lines were compared between HDAC inhibitor-treated cells and untreated cells. As shown in Figure 7, cell migration was inhibited in a dose-dependent manner by different concentrations of TSA, with up to 78.64% and 69.98% at 200 nM and 400 nM TSA incubation, respectively. Sodium butyrate showed similar inhibitory effects of migration on corneal fibroblasts (data not shown).

### HDAC inhibitors induced the senescence-like state of corneal myofibroblasts

**Figure 8:**
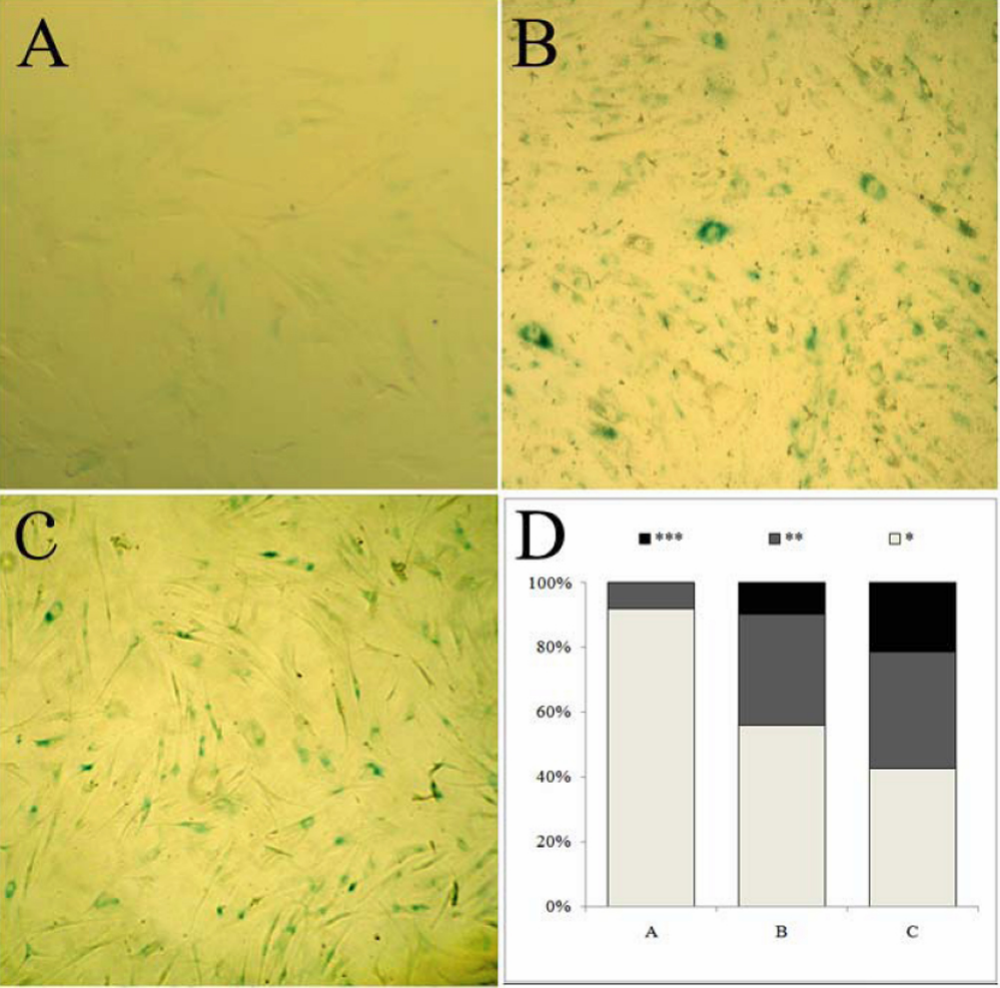
TSA induced the senescence-like state of corneal fibroblasts as indicated by β-galactosidase staining. TSA at 400 nM induced reduced saturation densities in corneal fibroblasts and more prominent β-galactosidase activity (**B**) than the untreated cells of equal density (**A**), while the positive control cells (treated with 100µM H_2_O_2_) showed enhanced staining of β-galactosidase (**C**). **D**: Image quantitative analysis of different β-galactosidase staining densities; the number of asterisks represents different densities with the three astrisks indicating the strongest staining of β-galactosidase.

**Figure 9:**
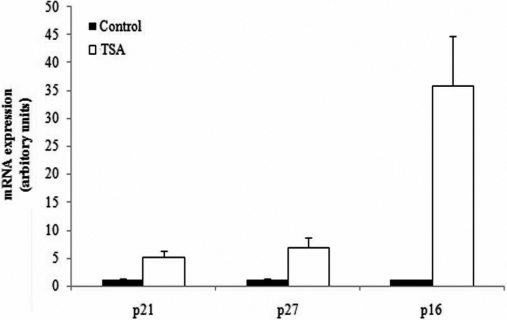
TSA caused the upregulation of the expressions of cell cycle-related transcriptional factors and biochemical markers of cell senescence. Compared with the untreated cells, the expressions of p16^ink4a^, p27^kip1^ and p21^cip1/waf1^ were upregulated by 35.85, 6.98, and 5.15 fold, respectively, by TSA.

Senescent cells can accumulate senescence-associated β-galactosidase activity that distinguishes them from quiescent cells [24]. We found that TSA induced corneal myofibroblasts showed reduced saturation densities and more prominent β-galactosidase activity than the control cells of equal density (Figure 8). Furthermore, the results of real time PCR for p16^ink4a^, p27^kip1^ and p21^cip1/waf1^, the cell cycle-related transcriptional factors and biochemical markers of cell senescence [3], revealed that treatment with TSA promoted their expressions in human corneal fibroblasts by 35.98, 6.98 and 5.15 fold, respectively, compared with the untreated cells (Figure 9). Taken together, the results demonstrated that HDAC inhibitors also induced the senescence-like state of corneal myofibroblasts, except for the inhibition of myofibroblastic differentiation and cell proliferation and migration.

## Discussion

**Figure 10:**
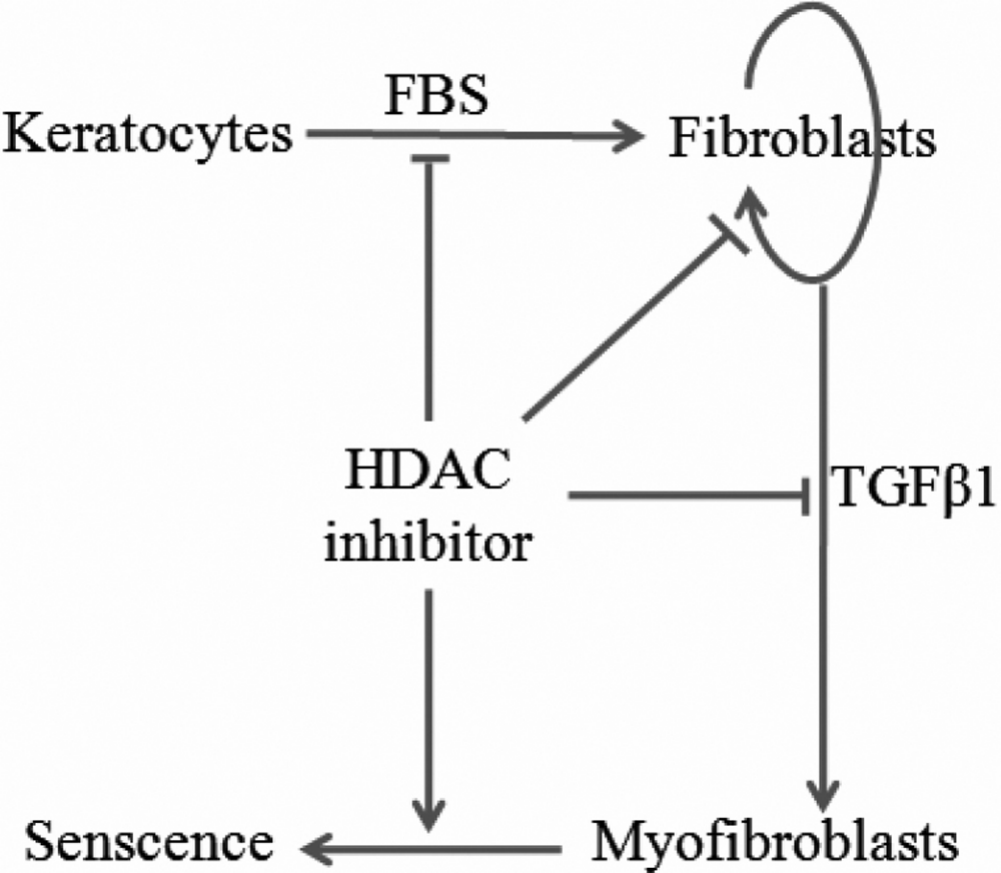
Schematic diagram of multiple effects of HDAC inhibitors on corneal stromal cells. HDAC inhibitors inhibited myofibroblastic differentiation of corneal stromal cells, repressed cell proliferation and migration of corneal fibroblasts, and induced cell senescence of corneal myofibroblasts.

Mounting data confirms that activated corneal stromal cells play an important role in corneal wound healing [1]. The formation of fibrotic stromal scars has often been described after refractive surgery or severe ocular injury, which is characterized by the increasing density of corneal fibroblasts, the appearance of corneal myofibroblasts and the disorganized corneal stromal matrix [25,26]. In the present study, we found that (1) HDAC inhibitors strongly inhibited the activation and myofibroblastic differentiation of corneal stromal cells, including morphological changes, expression of collagens and α-SMA, and the distribution of stress fibers, (2) HDAC inhibitors inhibited the proliferation of corneal fibroblasts by decreasing the proportion of cells in S-phase and increasing the proportion of cells in the G_0_/G_1_ and G_2_/M cell cycle checkpoints, (3) HDAC inhibitors repressed the migration of corneal fibroblasts in a dose-dependent manner, and (4) HDAC inhibitors induced the senescence state of corneal myofibroblasts (Figure 10).

HDAC inhibitors suppressed the morphological changes of corneal stromal cells induced by serum and TGFβ as their roles in hepatic and pancreatic stellate cells and skin fibroblasts [5,7,8]. Specifically for the mouse cells, treatment with HDAC inhibitors maintained the original morphology of corneal stromal cells in vivo. Along with the inhibitory effects on morphological changes, TSA-treated cells down-regulated the expressions of collagens and α-SMA mRNA and protein to their basal levels. The mechanisms of inhibitory effects of TSA on the myofibroblastic differentiation of corneal stromal cells need to be further elucidated. Inhibitory effects of HDAC inhibitors on cell proliferation and migration has been described in both normal and transformed cells [27,28]. Here, we found that 400 nM TSA or 5 mM NaBu decreased the proportion of cells in S-phase and increased the proportion of cells in the G_0_/G_1_ and G_2_/M cell cycle checkpoints. Furthermore, the expression of p21^waf1/cip1^ and p27^kip1^, the two cell cycle cyclin-dependent kinase (CDK) inhibitors associated with G_0_/G_1_ and G_2_/M cell cycle arrests, were upregulated after the treatment with TSA. Thus, we conclude that the upregulated expression of p21^waf1/cip1^ and p27^kip1^ induced by HDAC inhibitors may, at least partly, be responsible for the inhibition of proliferation and the cell cycle arrest effects of corneal fibroblasts. For the evaluation of migration inhibitory effects, wound healing assay were used. Because of the fibroblastic properties, we counted the number of cells migrated between the two injury lines instead of the measurement of wound width. The results showed that TSA inhibited the migration of corneal fibroblasts in a dose-dependent manner. However, the difference became significant when the cells were treated with the concentration of TSA above 100 nM.

In the analysis of real time PCR, we observed surprisely that, compared with the untreated human corneal myofibroblasts, 400 nM TSA increased the expression of p16^ink4a^ by about 36-fold, which is a well known marker of cell senescence [29,30]. So we hypothesized that HDAC inhibitors could induce the senescence-like state of corneal myofibroblasts, except for the inhibition of myofibroblastic differentiation and cell proliferation and migration. To further confirm this hypothesis, we tested the activity of the senescence associated β-galactosidase (SA-β-Gal) that distinguishes the senescent cells from quiescent cells [31,32]. As shown by the staining of SA-β-Gal, TSA-stimulated human corneal myofibroblasts expressed enhanced levels of SA-β-Gal activity. Furthermore, from the results of microarray analysis, we observed that the expression of other well known markers of replicative senescence was upregulated, such as MMP1 by 3.52 fold, while IGFBP2, IGFBP4 and IGFBP5 were upregulated by 8.12, 2.20, and 4.03 fold, respectively. However, IGFBP3 was downregulated by 0.39 fold by HDAC inhibitors in the cDNA microarray analysis (data not shown), which was consistent with previous reports that showed that the expressions of IGFBP4 and IGFBP5 were increased in senescent cells, while the IGFBP3 expression declined when the cells were completely senescent [29,33].

**Figure 11:**
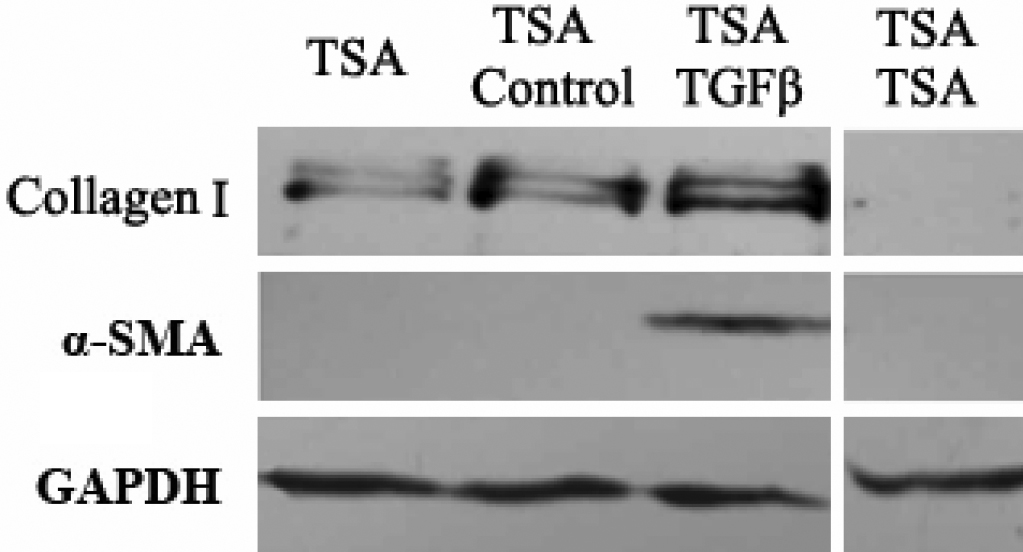
Analysis of partially reversible inhibitory effects of TSA on the myofibroblastic differentiation of corneal stromal cells. The samples were prepared as the corneal fibroblasts treated with 400 M TSA for 4 days (marked as the TSA lane), cells treated with 400 M TSA for 4 days and further incubation in the control medium (marked as the TSA-Control lane) or in the presence of TGFβ1 (marked as the TSA-TGFβ1 lane), cells treated with 400 M TSA for 8 days (marked as the TSA-TSA lane). The expression of collagen I and α-SMA were up-regulated after the removal of TSA of 4 days, and even rescued from the inhibition of HDAC inhibitors in the presence of TGFβ1.

It should be mentioned that the inhibitory effects of HDAC inhibitors may be partially reversible as previous descriptions [34-36], including the myofibroblastic differentiation, proliferation and migration, and the senescence-inducing effects on corneal stromal cells. As showed in Figure 11, the expression of collagen I and α-SMA were upregulated after the removal of TSA of 4 days, and even rescued from the inhibition of HDAC inhibitors in the presence of TGFβ1. In conclusion, the inhibitory effects of HDAC inhibitors on myofibroblastic differentiation of corneal stromal cells could be reversed by the persistent existence of TGFβ, but the overall anti-fibrotic effects of HDAC inhibitors on corneal scar formation should be evaluated in vivo.

In conclusion, we confirmed the inhibitory effects of HDAC inhibitors on corneal stromal cells were regulated by multiple mechanisms, including the inhibition of myofibroblastic differentiation of corneal stromal cells, the suppression of proliferation and migration of corneal fibroblasts and the induction of cell senescence of corneal myofibroblasts. Thus, further investigation of HDAC inhibitors may provide insights for developing promising drugs for the prevention or treatment of corneal haze and scar formation.
